# Bilateral middle cerebellar peduncle lesions: Neuroimaging features and differential diagnoses

**DOI:** 10.1002/brb3.1778

**Published:** 2020-08-05

**Authors:** Jiwei Jiang, Jirui Wang, Meiqing Lin, Xiaoting Wang, Jinli Zhao, Xiuli Shang

**Affiliations:** ^1^ Department of Neurology The First Affiliated Hospital of China Medical University Shenyang City Liaoning Province China; ^2^ Department of Radiology The First Affiliated Hospital of China Medical University Shenyang City Liaoning Province China

**Keywords:** magnetic resonance imaging, middle cerebellar peduncle, multiple system atrophy, neuromyelitis optica, stroke, Wallerian degeneration

## Abstract

**Objectives:**

Lesions limited to the bilateral middle cerebellar peduncles (MCPs) are uncommon. This retrospective study investigated diseases with a proclivity for the bilateral MCPs and explored the associations between their neuroimaging features and clinical findings for the differential diagnosis of such lesions.

**Methods:**

We enrolled 26 patients who were admitted to our department between January 2016 and March 2019 with bilateral MCP abnormalities on magnetic resonance imaging (MRI). The demographic, clinical, and neuroimaging characteristics, and the biomarkers and diagnoses were evaluated.

**Results:**

Although all patients exhibited symmetrical bilateral MCP hypointensities on T1‐weighted imaging and hyperintensities on T2‐weighted and fluid‐attenuated inversion recovery imaging, they were diagnosed with different conditions. Diagnoses included acute cerebral infarction (ACI) (*n* = 9, 34.62%), Wallerian degeneration (WD) (*n* = 8, 30.77%), multiple system atrophy (MSA) (*n* = 6, 23.08%), neuromyelitis optica (NMO) (*n* = 1, 3.85%), heroin‐induced leukoencephalopathy (*n* = 1, 3.85%), and primary central nervous system lymphoma (PCNSL) (*n* = 1, 3.85%). Patients with ACI exhibited bilateral MCP‐restricted diffusion hyperintensities on diffusion‐weighted imaging and corresponding stenosis or occlusion of the vertebrobasilar system. The initial MRI of patients with WD depicted pontine infarctions, while symmetrical MCP lesions were observed on follow‐up MRI. Symmetrical MCP lesions, cruciform hyperintensity, and marked atrophy in the posterior fossa were characteristic manifestations of MSA. Longitudinally extensive myelitis affecting more than three vertebral segments on cervical MRI and positive serum AQP4‐IgG may be indicative of NMO. Heroin‐induced leukoencephalopathy was characterized by extra‐symmetrical lesions in the posterior limbs of the internal capsules, while the anterior limbs were spared. PCNSL was indicated by a significant and characteristic “fist” sign on contrast‐enhanced MRI.

**Conclusions:**

Bilateral MCP lesions were most frequently observed in cerebrovascular diseases, followed by neurodegenerative diseases, inflammatory diseases, toxic encephalopathies, and lymphomas. Our findings demonstrate that bilateral MCP signal abnormalities are more common in patients with ACI and WD, with fewer degenerative processes than previously believed. The high frequency of WD may be attributed to the specific awareness of this pathology. WD can also present with stage‐related restricted diffusion and should not be mistaken for a new infarction. The symmetrical bilateral MCP hypointensities on T1‐weighted imaging and hyperintensities on T2‐weighted imaging often raise concern regarding a demyelinating process. Our findings emphasize that neurologists should consider the aforementioned conditions and correlate the specific neuroimaging characteristics and medical history before arriving at the final diagnosis.

## Graphical abstract

We report a retrospective case series of 26 patients with bilateral middle cerebellar peduncle (MCP) lesions and compared the differences in clinical presentations and neuroimaging features of their associated diseases. Our findings showed that symmetrical bilateral MCP lesions occurred in various pathologic conditions, which were most frequent in cerebrovascular diseases, followed by neurodegenerative diseases, inflammatory diseases, toxic encephalopathies, and lymphoma, and the features differed in manners that could aid in distinguishing among the diseases.

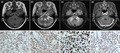

## 1 INTRODUCTION

The middle cerebellar peduncle (MCP), also called the brachium pontis, is the largest afferent system of the cerebellum. It consists of pontocerebellar tract (PCT) fibers arising from the contralateral pontine nuclei (Perrini, Tiezzi, Castagna, & Vannozzi, 2013). The detection rate of posterior fossa lesions has improved with rapid advancements in neuroimaging, and abnormal signals involving the MCPs on magnetic resonance imaging (MRI) have been recently described (Hall, Fraint, & Dafer, 2018; Polat, 2019). However, symmetrical lesions limited to the MCPs are rare. Although some studies have reported bilateral hyperintensities of the MCPs on T2‐weighted imaging in different diseases, their findings were not comprehensive (Okamoto, Tokiguchi, & Furusawa, 2003; Uchino, Sawada, Takase, & Kudo, 2004). Therefore, we retrospectively analyzed the data from 26 patients with bilateral MCP lesions on MRI and compared the clinical and neuroimaging features of different diseases, and correlated the imaging findings with clinical clues to aid differential diagnosis.

## 2 MATERIALS AND METHODS

### 2.1 Patient selection

This retrospective study enrolled 34 consecutive patients with bilateral MCP lesions treated in the Department of Neurology at the First Affiliated Hospital of China Medical University, a comprehensive academic hospital in a large urban area, between January 2016 and March 2019. We used the terms “bilateral MCP” and “bilateral brachium pontis” to retrieve data from the hospital's electronic information database system, which includes the information of a large number of patients who were hospitalized at our center. Patients were excluded from the study if they did not meet the respective diagnostic criteria (outlined below) or did not undergo radiological examination with MRI. Eight patients were excluded from the study based on these criteria. Twenty‐six patients were included in the final review. This study was approved by the appropriate ethics review board.

### 2.2 Clinical information and diagnostic criteria

The clinical information obtained included demographics (age of onset and sex), neurological signs and symptoms, disease‐specific biomarkers (e.g., anti‐aquaporin‐4 [AQP4]‐IgG), neuroimaging findings, and diagnoses. One patient underwent brain biopsy.

The diagnosis of each condition was based on its clinical diagnostic criteria, as follows. (a) Patients with acute cerebral infarction (ACI) met the Baltimore‐Washington Cooperative Young Study Criteria, including neurological deficit lasting longer than 24 hr and MRI showing infarctions corresponding to their clinical findings, while those with transient ischemic attack or cerebral hemorrhage were excluded (Johnson, Kittner, & McCarter, 1995) (b) Patients with Wallerian degeneration (WD) were identified by the presence of initial pontine damage with subsequent abnormal bilateral MCP findings on MRI; moreover, they presented with clear fiber tract connections between the pontine and MCP lesions (De Simone, Regna‐Gladin, Carriero, Farina, & Savoiardo, 2005; Shen et al., 2018). (c) Patients with multiple system atrophy (MSA) met the criteria for probable MSA detailed in the second consensus statement on the diagnosis of MSA: a sporadic, progressive, adult‐onset disease characterized by autonomic failure and poorly levodopa‐responsive parkinsonism or a cerebellar syndrome (Gilman, Wenning, & Low, 2008) (d) Patients with neuromyelitis optica (NMO) met the international consensus diagnostic criteria for NMO spectrum disorder (NMOSD) (Wingerchuk, Banwell, & Bennett, 2015): the core clinical characteristics, including optic neuritis and acute myelitis, a positive test for AQP4‐IgG, and the exclusion of alternative diagnoses. (d) Heroin‐induced leukoencephalopathy was diagnosed by a positive heroin test, acute intoxication typically presenting as classic toxic leukoencephalopathy, supporting radiological abnormalities in the bilateral periventricular white matter or the cerebellum, and the exclusion of alternative diagnoses (Alambyan, Pace, & Miller, 2018), (e) Patients with primary central nervous system lymphoma (PCNSL) met the guidelines for the diagnosis and management of primary central nervous system diffuse large B‐cell lymphoma: stereotactic biopsy and contrast‐enhanced brain MRI (Fox, Phillips, & Smith, 2019).

### 2.3 Magnetic resonance imaging

Images were obtained using 1.5‐T/3.0‐T magnetic resonance devices (Magnetom H‐15 and Vision, Siemens, Erlangen, Germany) by two experienced radiologists. Conventional axial brain MRI images were available for all patients. Axial T1‐weighted spin‐echo (SE) (repetition time/echo time: 500–550/9–15), T2‐weighted (4000–5000/96–100), and fluid‐attenuated inversion recovery (FLAIR) (8000–9000/94–100) imaging examinations were performed with echo train lengths of 5. Other MRI scan parameters included section thickness, 5 mm; field of view, 240 × 240 mm; and matrix, 320 × 320. Diffusion‐weighted imaging (DWI) (b = 0/1000 s/mm^2^, 6000/80–90, matrix, 192 × 192) and apparent diffusion coefficient (ADC) maps were acquired for 20 patients. Five patients underwent contrast‐enhanced MRI after intravenous (IV) administration of gadobutrol (0.1 mmol/kg), while 17 patients underwent computed tomography angiography (CTA) with ioversol (1 ml/kg) or MR angiography (MRA). Sagittal cervical and thoracic spinal MRIs were available for two patients with T1‐weighted SE (500–550/10–15) and T2‐weighted SE (3000–4000/100–120).

## 3 RESULTS

### 3.1 Clinical findings

The demographics, specific symptoms/signs, neuroimaging findings, relevant biomarkers, and final diagnoses are summarized in Table 1. ACI was the most common diagnosis (*n* = 9, 34.62%), followed by WD (*n* = 8, 30.77%), MSA (*n* = 6, 23.08%), NMO, heroin‐induced leukoencephalopathy, and PCNSL (*n* = 1, 3.85% each).

**TABLE 1:** Summary of the clinical presentations and radiological findings of 26 cases

Diagnosis (n)	Onset age (y)/gender			MRI			Other imaging features	Special presentations and examinations
		T1	T2	FLAIR	DWI	ADC		
ACI (9)	65,59,86,54,68/M	L	H	H	H	L	**involved vessels (n)**	sudden onset with duration of corresponding neurological function deficits lasting over 24 hours
	74,62,77,76/F						basilar artery (4)	
							vertebral artery (4)	
							AICA (6)	
							SCA (2)	
WD (8)	64,63/M; 69/F	L	H	H	H	H	**initial infarction (n)** bilateral pontine (2) left pontine (3) right pontine (3)	all patients without sudden‐onset neurological symptoms
	56/M	L	H	H	H	L		
	51/F	L	H	H	H	—		
	59,49,53/M	L	H	H	N	N		
MSA‐C (6)	54,60/M	L	H	H	H	H	“hot cross bun” sign (2/6) only a vertical line (4/6) atrophy of the cerebellum and brainstem	progressive gait ataxia with cerebellar dysarthria, limb ataxia or cerebellar oculomotor dysfunction, unexplained urinary abnormality or orthostatic hypotension
	59,61,44,63/F							
					
NMO (1)	46/F	L	H	H	—	—	extensive spinal cord lesion from C3 to C6 on cervical MRI	four consecutive attacks of bilateral optic neuritis and acute myelitis with positive serum AQP4‐IgG
Heroin‐induced leukoencephalopathy (1)	49/F	L	H	H	H	—	symmetrical lesions on the posterior limbs of the internal capsules	heroin inhalation history, altered level of consciousness (from confusion to coma)
PCNSL (1)	54/F	L	H	H	—	—	patchy enhancement signals	biopsy confirmed diffuse large B‐cell lymphoma

Abbreviations: —, not obtained; ACI, acute cerebral infarction; AICA, anterior inferior cerebellar artery; AQP4‐IgG, anti‐aquaporin‐4 IgG; H, high signal; L, low signal; MRI, magnetic resonance imaging; MSA‐C, multiple system atrophy‐cerebellar variant; *N*, normal; *n*, number; NMO, neuromyelitis optica; PCNSL, primary central nervous system lymphoma; SCA, superior cerebellar artery; WD, Wallerian degeneration; y, years.

### 3.2 Magnetic resonance imaging findings

**FIGURE 1:**
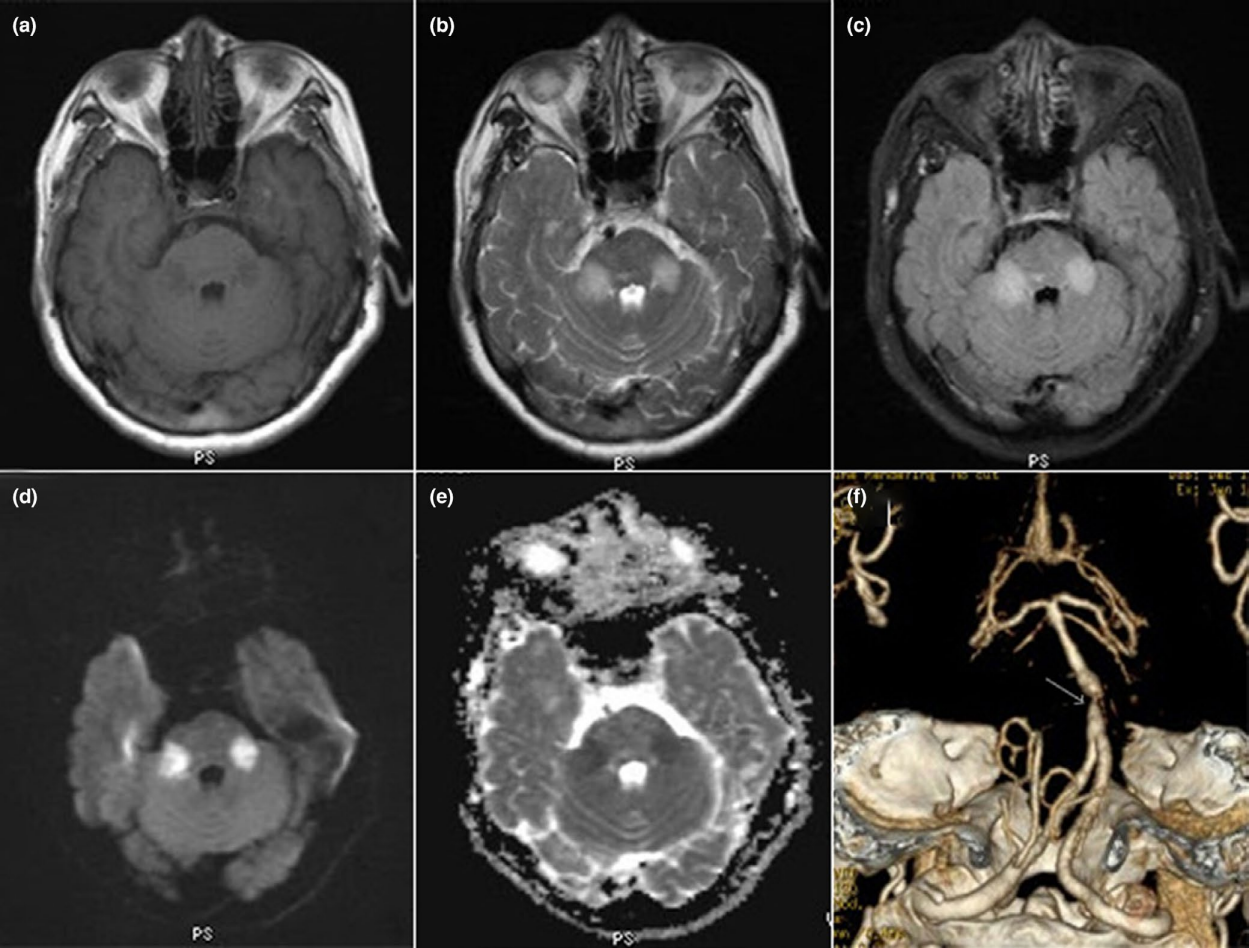
A 31‐year‐old woman with AQP4‐IgG‐seropositive neuromyelitis optica. Axial brain T1‐weighted imaging (a) shows hypointensity in both MCPs, and hyperintensity on T2‐weighted (b) and fluid‐attenuated inversion recovery imaging (c). Axial diffusion‐weighted imaging (d) demonstrates restricted diffusion signals in both MCPs, with corresponding hypointensity on the apparent diffusion coefficient map (e). Computed tomographic angiography (f) reveals severe stenosis of the basilar artery near the origin of the anterior inferior cerebellar artery (AICA; white arrow) and bilateral AICA occlusion

**FIGURE 2:**
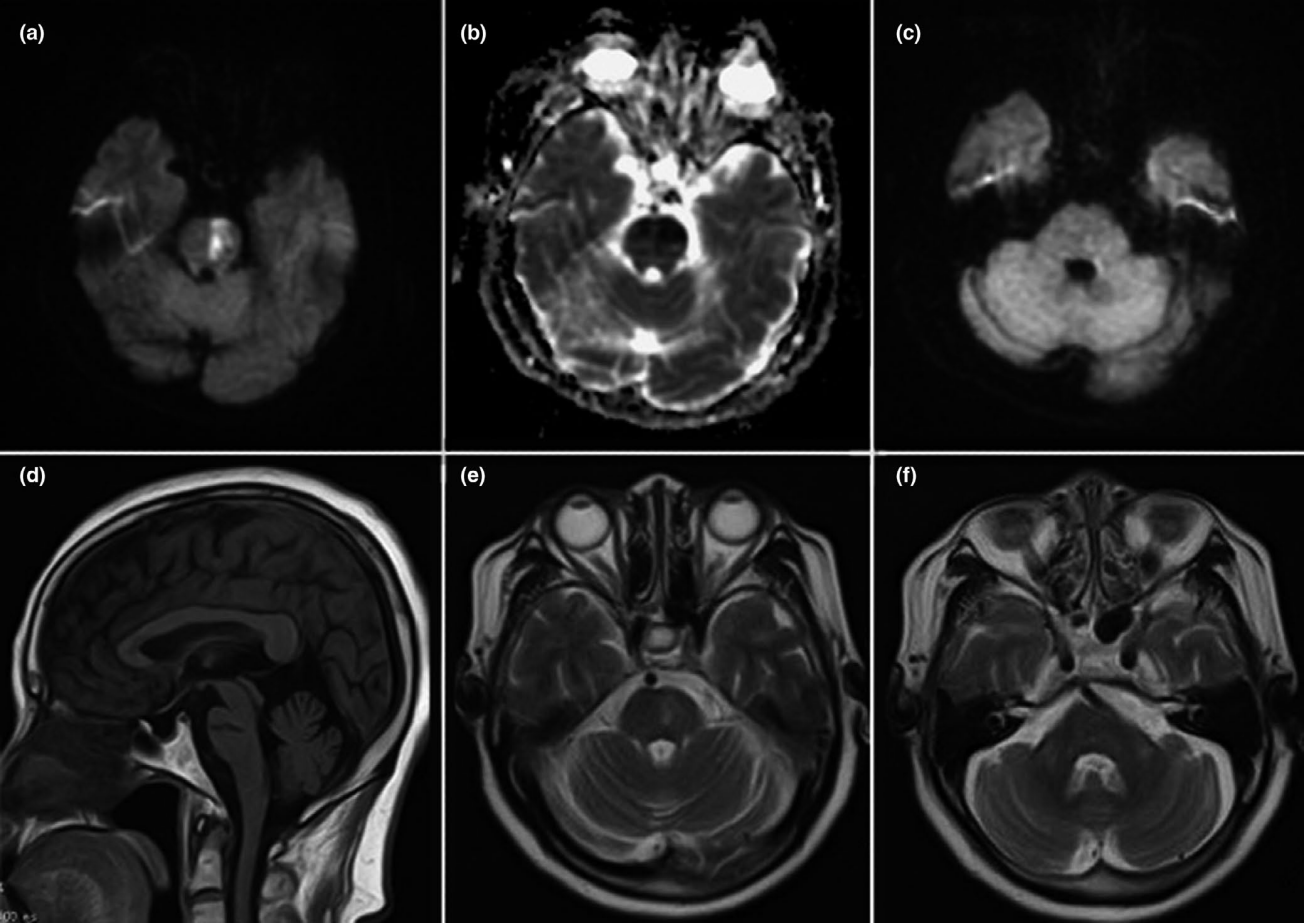
A 69‐year‐old woman with Wallerian degeneration after pontine infarction. Initial diffusion‐weighted imaging shows restricted diffusion of the left pons (a), with corresponding hypointensity on the apparent diffusion coefficient map (b) consistent with acute infarction. At this time, no MCP lesion is visible (c). On follow‐up MRI 4 months later, hypointensity on sagittal T1‐weighted imaging (d) and hyperintensity on pontine T2‐weighted imaging (e) indicates a previous infarction. Symmetrical hyperintensity of both MCPs on T2‐weighted imaging (f) suggests Wallerian degeneration of the pontocerebellar fibers

**FIGURE 3:**
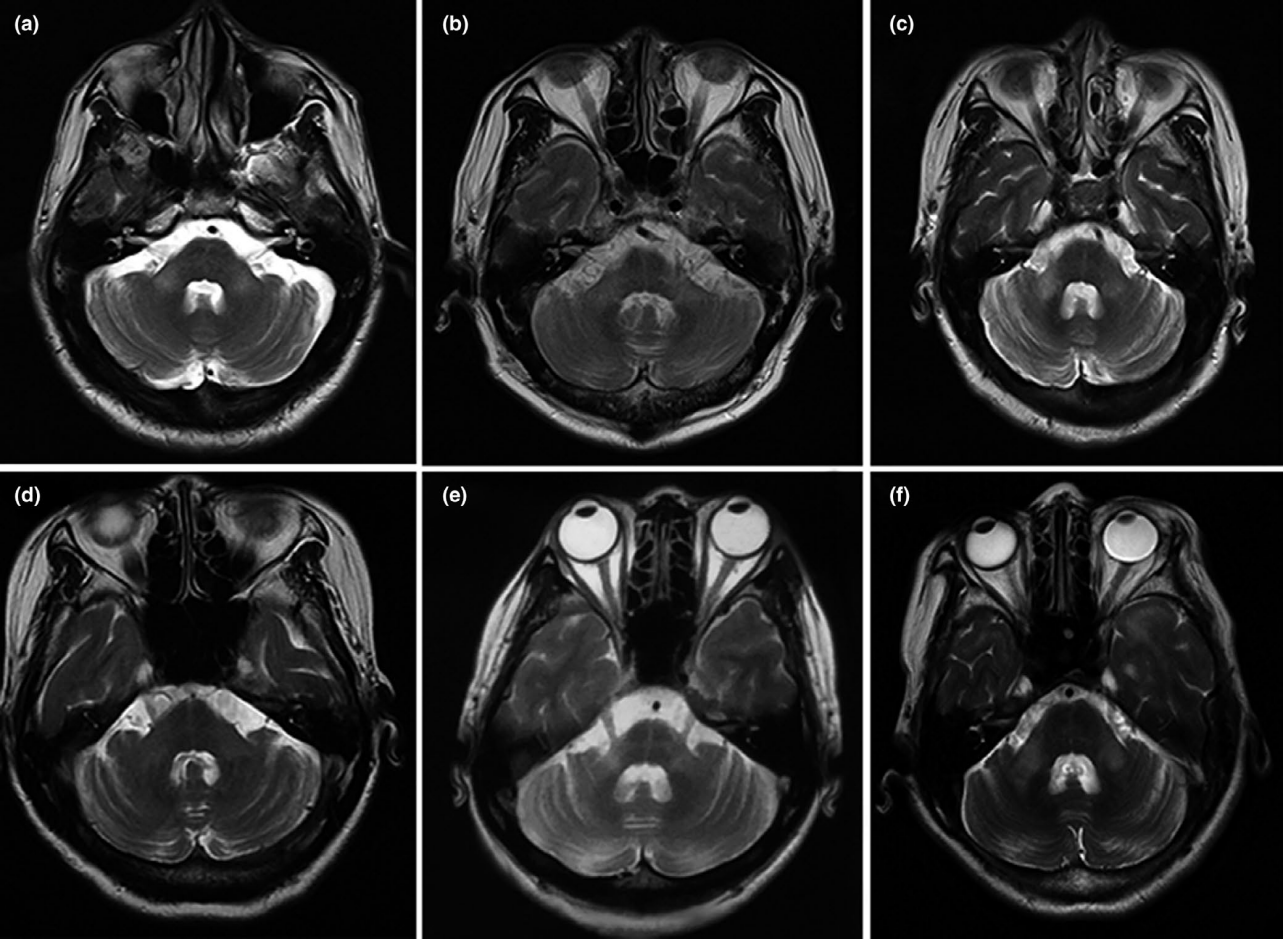
Brain MRI of six patients with multiple system atrophy. All patients show an increasing signal of both MCPs on T2‐weighted imaging. Axial pontine T2‐weighted imaging shows a clear vertical line and a fuzzy horizontal line of hyperintensity (a,c,d,f), and both clear horizontal and vertical lines of hyperintensity (“hot cross bun” sign) with mild atrophy of the brainstem, MCPs, and cerebellum (b,e)

**FIGURE 4:**
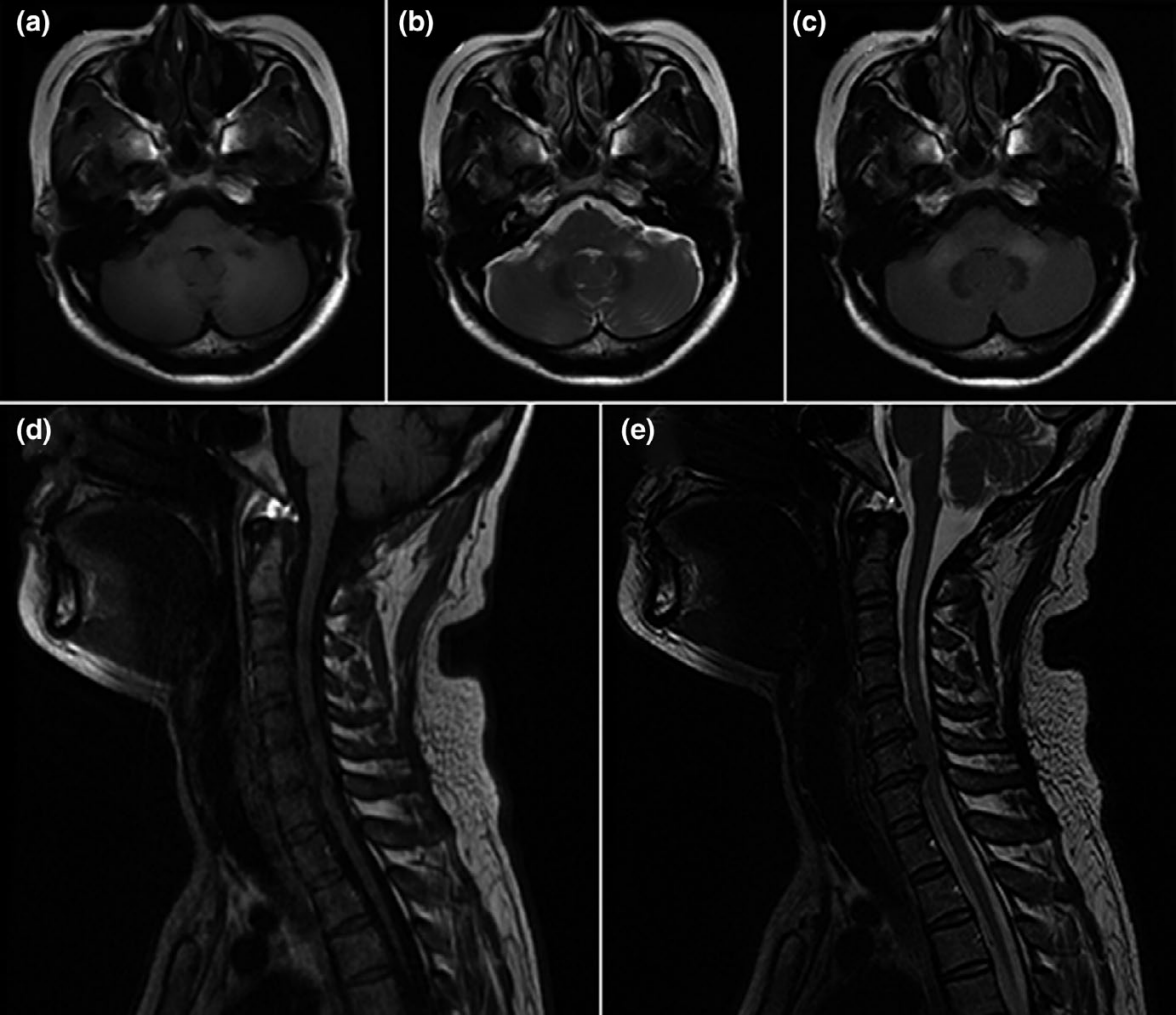
A 31‐year‐old woman with AQP4‐IgG‐seropositive neuromyelitis optica. Axial brain T1‐weighted imaging (a) shows hypointensity in both MCPs, with hyperintensity on T2‐weighted (b) and fluid‐attenuated inversion recovery (c) imaging. Sagittal T1‐weighted imaging (d) of the cervical spinal cord shows central linear hypointensity extending from C3 through C6, with high signal intensity on T2‐weighted imaging (e)

**FIGURE 5:**
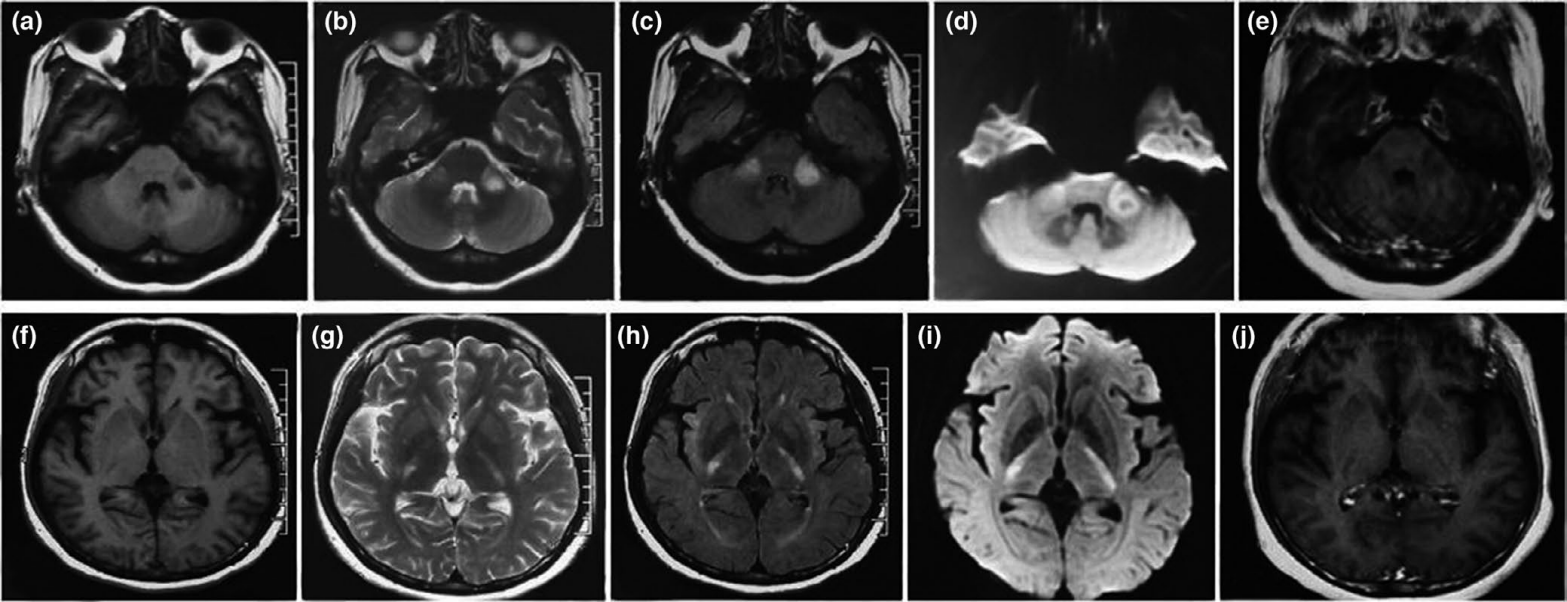
A 49‐year‐old woman with heroin‐induced leukoencephalopathy. Axial brain T1‐weighted imaging shows low signal intensity affecting the bilateral MCPs (a) and optic radiations, as well as the posterior limbs of the internal capsules, with sparing of the anterior limbs (f) and corresponding hyperintensity on T2‐weighted (b,g), fluid‐attenuated inversion recovery (c,h), and diffusion‐weighted imaging (d,i). Enhanced scanning does not demonstrate significant enhancement in both the MCPs and internal capsules (e,j)

**FIGURE 6:**
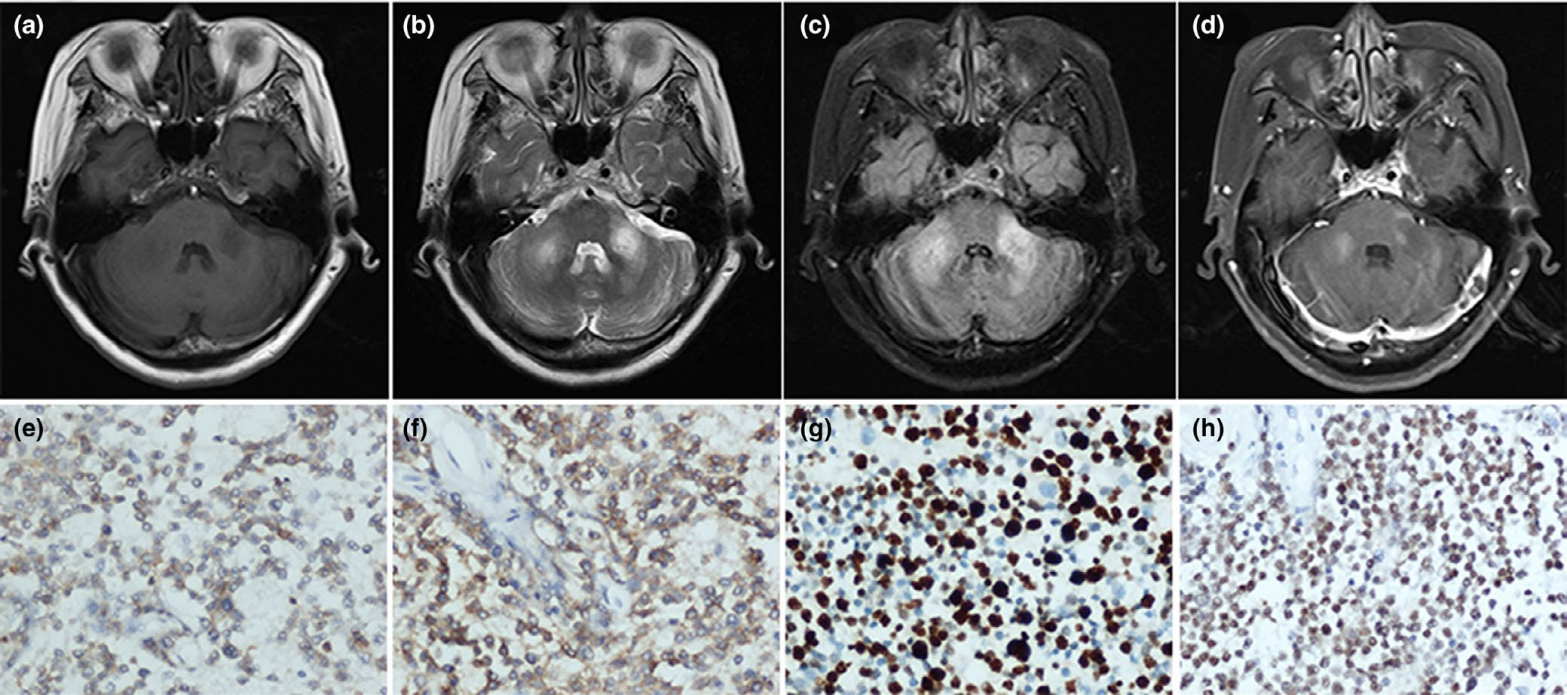
A 54‐year‐old woman with primary central nervous system lymphoma. Axial brain T2‐weighted (b) and fluid‐attenuated inversion recovery (c) imaging show ill‐defined hyperintensity with a minimal associated mass effect involving the bilateral MCPs, with hypointensity on T1‐weighted imaging (a). Contrast‐enhanced T1‐weighted MRI demonstrates abnormal patchy enhancement at the same location (d). Immunohistochemistry findings are CK^−^, CD3^+^, CD20^+^ (f), Pax‐5^+^ (h), Bcl2^+^, CD21^−^, CD23^−^, CD10^−^, CD5^+^ (e), CyclinD1^−^, CD30^−^, and Ki‐67(80%+) (g), consistent with diffuse large B‐cell lymphoma

All patients had bilateral symmetrical MCP hypointensities on T1‐weighted imaging and hyperintensities on T2‐weighted and FLAIR imaging. However, there were major differences in other neuroimaging findings for the different disorders (Table 1). Figure 1 describes a patient with isolated bilateral MCP infarctions and basilar artery (BA) occlusion. Eight of nine patients with ACI underwent angiography (five CTA, two MRA, and one digital subtraction angiography) of the involved vessels (Table 1). WD presented as an initial acute pontine infarction on initial MRI, with abnormal signals of the bilateral MCPs on follow‐up MRI (Figure 2). However, the characteristics of the initial infarctions and signals on DWI and ADC showed changes in the subsequent stages of WD in different patients (Table 1). All patients with MSA were diagnosed with the cerebellar variant (MSA‐C). Two patients had a clear and complete “hot cross bun” sign in the pontine, with atrophy of the cerebellum and brainstem, while only a clear vertical line was observed in four patients (Figure 3). Figure 4 shows a patient with NMO who presented with bilateral MCP lesions with intramedullary hyperintensity extending over three contiguous segments on cervical T2‐weighted imaging. The brain MRI of the patient with heroin‐induced leukoencephalopathy revealed extra‐symmetrical abnormal signals in the optic radiations and posterior limbs of the internal capsules, while the anterior limbs were spared (Figure 5). Symmetrical patchy enhancement masses of the bilateral MCPs were observed on enhanced MRI for PCNSL (Figure 6).

## 4 DISCUSSION

Isolated bilateral abnormalities of the MCPs on brain MRI are rare. Previous studies have indicated that neurodegenerative diseases are most likely to affect both MCPs (Morales & Tomsick, 2015; Okamoto et al., 2003). However, bilateral MCP lesions were signs of other diseases in this study, the most common being ACI, followed by WD, MSA, NMO, heroin‐induced leukoencephalopathy, and PCNSL. Despite reports on all these pathologies mentioned previously, to the best of our knowledge, NMO and PCNSL lesions were not restricted to the bilateral MCPs but were often accompanied by other cerebral lesions in previous studies. We think that the differences in the frequency of disease distribution observed in our study, especially the increase in incidence of ACI and the decrease in that of degenerative processes, are related to the inclusion criteria or selection bias. Moreover, we attribute the high frequency of WD, which was not reported in the Okamoto review, to an increase in the specific awareness of this pathology (Okamoto et al., 2003). Although these patients had similar abnormal signals in the bilateral MCPs on conventional MRI, resulting in similar clinical symptoms/signs, there were major differences in their medical histories, other iconic imaging findings, and biomarkers. This study aimed to discuss the possible mechanisms underlying bilateral MCP lesions and explore the associations between neuroimaging features and clinical clues to facilitate differential diagnosis.

Isolated infarction of the bilateral MCPs is rare. We reported nine patients with primary infarctions limited to the bilateral MCPs due to large‐artery atherosclerosis, which represents the most extensive series of these patients, to the best of our knowledge. Significant anatomical variations exist in the vasculature supplying the MCPs. Typically, the anterior inferior cerebellar artery (AICA) diverges from the BA of origin and serves as the main vascular supply of the MCPs. Anatomical variations are most commonly seen as caudal AICA atresia or rostral duplicate AICA occlusion (Chen, Chen, Diaz‐Marchan, Schomer, & Kumar, 2018). Moreover, the territory perfused by the AICA may overlap with that of the marginal branch of the superior cerebellar artery (SCA); thus, the MCPs could be supplied by the SCA directly or by a watershed zone supplied by both vascular territories in patients with an AICA variation (De Cocker, Lövblad, & Hendrikse, 2017; Kataoka, Izumi, & Kinoshita, 2011; Rhoton, 2000). Moreover, some studies reported that bilateral MCP infarctions could be attributed to varying degrees of vertebral artery occlusion or stenosis (De Cocker et al., 2017; John, Hegazy, Cheng Ching, & Katzan, 2013). Therefore, alterations in the vertebrobasilar system, especially the AICA, should be considered as a possible cause of isolated bilateral MCP infarctions.

WD refers to progressive anterograde disintegration with demyelination of the distal axons following injury to the proximal axon or soma (De Simone et al., 2005). Currently, there is no definitive clinical diagnostic standard for WD. However, the histologic and metabolic characteristics of the different stages of WD are correlated with specific findings on conventional MRI (De Simone et al., 2005). The first stage (within 20 days after injury) is characterized by disintegration of the axons and myelin sheaths without abnormal signals on conventional MRI. However, several studies have revealed that DWI can depict transient signal abnormalities at this stage, especially within the first 2 weeks of the stroke (pre‐WD) (Castillo & Mukherji, 1999; Kang, Chu, & Yoon, 2000). The second stage (from 20 days to 2–4 months after injury) is characterized by rapid myelin protein breakdown, during which the tissue becomes more hydrophobic, resulting in hypointensities on proton‐density and T2‐weighted imaging. Myelin and lipid breakdown, gliosis, and increase in hydrophilic tissue during the third stage (14 weeks after injury) result in the appearance of hyperintensities on T2‐weighted and FLAIR imaging and hypointensities on T1‐weighted imaging. The last stage (after several years) is characterized by volume loss due to atrophy. Therefore, WD entails the degeneration of axonal structures and demyelination, and finally fibrosis and atrophy of the affected fiber tracts. WD is observed most frequently in the corticospinal tract (CST) (which contains numerous axons and executes essential functions) following injury to the motor cortex or internal capsule and presents as ipsilateral T2 hyperintensity or atrophy of the cerebral peduncle (Chen, Nabavizadeh, & Vossough, 2017). Recent studies have reported cases of WD of the bilateral MCPs secondary to pontine infarction, resulting from involvement of the PCT (De Simone et al., 2005; Shen et al., 2018). Thus, the MCPs are usually involved as they are the principal and largest pathways for the PCTs. PCT fibers arise from the contralateral pontine nuclei, cross the midline at the basis pontis, and pass through the MCPs to reach the cerebellar cortex; thus, WD of the MCPs often occurs bilaterally, even if the initial pontine lesion is unilateral (Uchino, Takase, Nomiyama, Egashira, & Kudo, 2006).

Consistent with the work of Shen et al. (Shen et al., 2018), our experience showed that WD of the PCT was easier to diagnose in the third stage because neurological deficits in patients with WD at this stage are more likely to appear in this period, and conventional MRI can detect abnormal signals. Notably, five of eight patients with WD in our study had mild bilateral MCP hyperintensities on DWI. Of them, one patient exhibited hypointensity (16 weeks after infarction) and three had slight hyperintensities (11, 16, and 17 weeks after infarction) on ADC, while information regarding the ADC sequence was unavailable for one patient (3 weeks after infarction). Castillo & Mukherji (1999) and Kang et al. (2000) have described several hyperintensities in CST during the early stage of WD (3–12 days). Shen et al. (2018) and Fitzek, Fitzek, & Stoeter (2004) have reported that increased signal intensities in MCPs on DWI can persist for 14–16 weeks after stroke. All the aforementioned observations are consistent with our findings, suggesting that diffusion abnormalities may occur in degenerating fibers and are time‐related, irrespective of the ADC sequence, especially during the first and third stages. This occurrence might be related to cell swelling, demyelination with axonal degeneration, phagocytotic activity, and water uptake and indicate that diffusion abnormalities in degenerating fibers are not specific and may provide evidence to differentiate WD from ACI. Therefore, neurologists should be familiar with WD of the bilateral MCPs to avoid misdiagnosis as an additional infarction.

The “hot cross bun” sign and atrophy of the posterior fossa, resulting from reduced histologic myelin staining of the transverse PCT fibers and the remaining CST, are the most recognized imaging characteristics of patients with MSA‐C (Chelban, Bocchetta, & Hassanein, 2019; Ngai, Tang, Du, & Stuckey, 2006). Horimoto et al. divided pontine cruciform T2 hyperintensity into six stages, wherein vertical and horizontal lines appear gradually on MRI, culminating in a “cross” sign with pontine atrophy. All patients showed a vertical line within 2–3 years after symptom onset; however, only 71% of cases showed the complete “hot cross bun” sign within 5 years (Horimoto, Aiba, & Yasuda, 2002). Moreover, the sensitivity of cruciform hyperintensity for MSA‐C is 50%, whereas that of the presence of MCP hyperintensity is 85% (Rizzo, Martinelli, & Manners, 2008; Schrag, Good, & Miszkiel, 2000). Consistent with these findings, our study suggests that a combination of these signs can contribute to the identification of MSA at different stages, and bilateral MCP abnormalities may be more sensitive to MSA identification compared to other infratentorial signs. As mentioned above, WD is a progressive process of degeneration and demyelination, which easily involves the fibers and MCPs (a massive bundle of fibers connecting the pons with the cerebellum). Moreover, neurodegenerative processes such as MSA (which also entails degeneration and demyelination) is well‐known for its predilection for MCPs. Thus, we emphasized the importance of the predilection of the two pathologic processes (degeneration and demyelination) for MCPs. This information may help clinicians evaluate such cases and formulate a differential diagnosis.

Recently, numerous researchers have found that brain abnormalities can be observed in several patients with NMOSD with an increase in disease duration, and that the detection of cerebral white matter lesions by MRI, indicating multiple sclerosis (MS), no longer excludes a diagnosis of NMOSD (Cabrera‐Gomez & Kister, 2012; Matthews & Palace, 2014). In 2015, the international consensus on the diagnostic criteria for NMOSD described relatively specific lesions in patients with NMOSD and brain abnormalities, including lesions in the dorsal medulla/area postrema, periependymal areas in the brainstem or diencephalic structures, and cerebral hemispheres, all of which are associated with a high AQP4 expression (Pittock et al., 2006; Wingerchuk et al., 2015). However, brain abnormalities were also observed in areas where the AQP4 expression was not particularly high, including extensive lesions spanning a considerable length of the corpus callosum and CST (Kim, Park, & Lee, 2010). Despite increasing knowledge of brain MRI characteristics in NMOSD, bilateral MCP lesions in patients with NMOSD‐related demyelination and inflammatory diseases are extremely rare, which makes them difficult to distinguish from atypical MS lesions, especially in AQP4‐IgG‐seronegative patients (Okamoto et al., 2003; Uchino et al., 2004). In such cases, longitudinally extensive transverse myelitis lesions spanning three or more vertebral segments, which is very rare in adult MS, could be another important and specific neuroimaging characteristic of NMOSD (Kim, Paul, & Lana‐Peixoto, 2015). Our patient experienced four consecutive attacks of bilateral optic neuritis and acute myelitis, was AQP4‐IgG‐seropositive, and had a contiguous intramedullary lesion that met the international consensus diagnostic criteria for NMOSD (Wingerchuk et al., 2015). It is interesting that, unlike the circumventricular areas, the PCTs do not have high AQP4 expression; nevertheless, we believe the unique PCT involvement extending from the brainstem to the MCPs may be similar to longitudinal brain abnormalities commonly involving the CST from the internal capsule to the cerebral peduncle, providing new evidence for symmetrical MCP T2 hyperintensities in patients with AQP4‐IgG‐seropositive NMO.

Toxic leukoencephalopathy is an uncommon but critical neurological disorder seen in heroin abusers. There are various routes of heroin misuse, including inhalation, IV injection, intranasal (snorting), and subcutaneous injection (Alambyan et al., 2018). “Chasing the dragon,” an inhalation method that involves heating heroin over aluminum foil and inhaling the resultant fumes, has recently become the most popular route of heroin intake due to its availability, greater ease of administration, and safer infectious profile compared to the other routes (Cicero, Ellis, Surratt, & Kurtz, 2014). A recent study showed that the brain MRIs of patients with heroin‐induced leukoencephalopathy differed greatly according to the intake routes (Cheng, Chin, & Chang, 2019): The MRI findings of patients who inhaled heroin were characterized by posterior to anterior involvement of the cerebral white matter and lesions in the posterior limbs of the internal capsules, cerebellum, and brainstem, whereas those of patients who injected heroin IV were characterized by lesions in the subcortical U‐fibers and genu of the internal capsule. The definitive mechanism underlying heroin‐induced leukoencephalopathy remains unclear. One neuropathological study suggested that it was characterized by spongiform vacuolar degeneration of the white matter due to apoptosis of oligodendrocytes and subsequent demyelination. (Yin, Lu, Chen, Fan, & Lu, 2013). Oligodendrocyte apoptosis‐induced demyelination in the white matter is more sensitive to ischemia/hypoxia because the axons and myelin sheaths in this area are thin (Yin et al., 2013). Therefore, in agreement with the pathological findings, brain MRI abnormalities caused by heroin inhalation often result in extensive, symmetrical lesions of the cerebral and cerebellar white matter, posterior limbs of the internal capsules, and splenium of the corpus callosum, which helps distinguish it from other causes of leukoencephalopathy such as toluene toxicity or reversible posterior leukoencephalopathy (Alambyan et al., 2018; Cheng et al., 2019; Offiah & Hall, 2008). Our patient, who had an extensive medical history of heroin inhalation with presentations ranging from confusion to coma, exhibited these specific bilateral symmetrical lesions in the posterior limbs of the internal capsules, while the anterior limbs were spared, consistent with the aforementioned observations. Moreover, symmetrical lesions involving the bilateral MCPs might be another novel neuroimaging feature in those who “chase the dragon.”

Contrast‐enhanced MRI is considered to be the best technique to detect and differentiate PCNSL, owing to the complexity of brain biopsy or cerebrospinal fluid analysis (Batchelor, 2019). A typical brain MRI in a patient with PCNSL shows a solitary, homogeneously enhancing mass or multiple lesions, frequently involving the frontal lobe, basal ganglia, corpus callosum, or periventricular white matter (Cheng & Zhang, 2019; Küker, Nägele, & Korfel, 2005). A significant and characteristic “incision,” “angular,” or “fist” sign, moderate edema, and the absence of necrosis is pathognomonic neuroimaging features (Patrick & Mohile, 2015). The presence of contrast enhancement in areas with abnormal signals in the MCP should evoke concern for a demyelinating process such as MS or progressive multifocal leukoencephalopathy, since lymphoma is a rare condition. To the best of our knowledge, lesions limited to the bilateral MCPs have not yet been reported in PCNSL. A good response to corticosteroids and atypical clinical presentations in patients with PCNSL may result in misdiagnosis as a demyelinating disease in those with isolated bilateral MCP lesions (Kerbauy et al., 2017). We believe that the characteristic imaging features of diffuse large B‐cell lymphoma should be reconsidered in immunocompetent patients. Our case of PCNSL provides new neuroimaging evidence for the diagnosis of PCNSL in immunocompetent patients.

This study had some limitations. First, it was a single‐center retrospective study with a small sample size. Second, the diffusion tensor imaging scans of some patients with degenerative diseases, which could have provided information on the structural integrity of the axonal white matter, were unavailable. Thus, future multi‐center studies with larger sample sizes and advanced MRI techniques may help strengthen the associations between bilateral MCP lesions and neurological diseases.

In conclusion, symmetrical bilateral MCP lesions occur in various pathological conditions, and our findings indicated they were most frequent in cerebrovascular diseases, followed by neurodegenerative diseases, inflammatory diseases, toxic encephalopathies, and lymphoma. Patients with these diseases may have similar clinical symptoms/signs and conventional brain MRI presentations. Hence, clinicians should consider additional specific neuroimaging characteristics while formulating a differential diagnosis. The definitive mechanism underlying the pathogenesis of bilateral MCP lesions remains unclear; however, the degeneration of transverse pontocerebellar fibers, as a part of diffuse white matter lesions or the continuous spread of pontic lesions, has been considered as the most probable cause. Prospective research, including basic studies and large clinical trials, is needed to elucidate the mechanisms underlying MCP‐related disorders and establish better guidelines for their differential diagnosis in clinical practice.

## CONFLICT OF INTEREST

The authors have no conflict of interest to declare.

## AUTHOR CONTRIBUTION

JJ: conceptualization and design of the study, interpretation of data, drafting and revising the manuscript. JW: critical revision of the manuscript for important intellectual content, ethics submission, and data analyses. ML: data collection and analyses. XW: ethics submission and data analyses. JZ: data collection and analyses. XS: critical revision of the manuscript for important intellectual content, study supervision, and fund support. All authors have read and approved the manuscript.

## ETHICAL STATEMENT

This study was approved by the appropriate ethics review board.

### Peer Review

The peer review history for this article is available at https://publons.com/publon/10.1002/brb3.1778.

## Data Availability

The data supporting the findings of this study are available from the corresponding author (Xiuli Shang) upon reasonable request.
